# Stimulatory Effects of Boron Containing Bioactive Glass on Osteogenesis and Angiogenesis of Polycaprolactone: In Vitro Study

**DOI:** 10.1155/2019/8961409

**Published:** 2019-03-18

**Authors:** Lunguo Xia, Wudi Ma, Yuning Zhou, Zhipeng Gui, Aihua Yao, Deping Wang, Akimichi Takemura, Mamoru Uemura, Kailin Lin, Yuanjin Xu

**Affiliations:** ^1^Department of Orthodontics, Ninth People's Hospital, Shanghai Jiao Tong University School of Medicine, Shanghai Key Laboratory of Stomatology, China; ^2^Department of Oral Surgery, Ninth People's Hospital, Shanghai Jiao Tong University School of Medicine, Shanghai Key Laboratory of Stomatology, China; ^3^Key Laboratory of the Advanced Civil Engineering Materials, Tongji University, Ministration of Education, China; ^4^Department of Anatomy, Osaka Dental University, Osaka, Japan; ^5^Department of Oral and Craniomaxillofacial Science, Ninth People's Hospital, Shanghai Jiao Tong University School of Medicine, Shanghai Key Laboratory of Stomatology, China

## Abstract

Polycaprolactone (PCL) has attracted great attention for bone regeneration attributed to its cost-efficiency, high toughness, and good processability. However, the relatively low elastic modulus, hydrophobic nature, and insufficient bioactivity of pure PCL limited its wider application for bone regeneration. In the present study, the effects of the addition of boron containing bioactive glass (B-BG) materials on the mechanical properties and biological performance of PCL polymer were investigated with different B-BG contents (0, 10, 20, 30, and 40 wt.%), in order to evaluate the potential applications of B-BG/PCL composites for bone regeneration. The results showed that the B-BG/PCL composites possess better tensile strength, human neutral pH value, and fast degradation as compared to pure PCL polymers. Moreover, the incorporation of B-BG could enhance proliferation, osteogenic differentiation, and angiogenic factor expression for rat bone marrow stromal cells (rBMSCs) as compared to pure PCL polymers. Importantly, the B-BG also promoted the angiogenic differentiation for human umbilical vein endothelial cells (HUVECs). These enhanced effects had a concentration dependence of B-BG content, while 30 wt.% B-BG/PCL composites achieved the greatest stimulatory effect. Therefore the 30 wt.% B-BG/PCL composites have potential applications in bone reconstruction fields.

## 1. Introduction

Biodegradable polymers and their copolymers are widely used in bone regeneration, such as polycaprolactone (PCL), lactic-co-glycolic acid (PLGA), and polylactic acid (PLA) [1–6]. Among these biodegradable polymers, PCL has attracted much attention attributed to its cost-efficiency, high toughness, and good processability resulting from its relatively low melting temperature [2, 5, 7–12]. However, the relatively low elastic modulus, hydrophobic nature, and insufficient bioactivity of pure PCL limited its wider applications for bone tissue regeneration [4, 13–16]. Thus far, a variety of inorganic phases have been applied as reinforcement for improving the mechanical properties and biological performance of PCL polymers.

It is well known that bioactive glasses (BGs) have been widely applied as scaffolds for bone tissue engineering and for in situ tissue regeneration, due to their excellent bone-forming bioactivity, biodegradation ability, and the positive biological effects of their reaction products on osteoblastic responses [17]. Moreover, the change in the composition of BGs by replacement and/or incorporation of different ions could regulate their bioactivity. As a trace element, boron (B) has been considered essential for bone physiology; it is reported that B deficiency resulted in the alteration or loss of important physiological functions associated with the metabolism of calcium and formation and remodeling of bone tissue [18–22]. Moreover, recent studies showed that incorporation of B in BGs (B-BG) could enhance osteogenesis in vitro and in vivo [23–28]. It is suggested that angiogenesis is important for bone regeneration in vivo [29, 30]. Previous studies reported that angiogenesis occurs before osteogenesis in the healing of bone defects, which could provide the blood supply and consequently benefit the subsequent progression of osteogenesis [29]. Consequently, both angiogenesis and osteogenesis participate in the process of bone regeneration and promote the effects of each other [31]. Moreover, recent studies showed that B-BG materials could stimulate cell proliferation, migration, and tube formation of human umbilical vein endothelial cells (HUVECs) and angiogenesis on the vasculature of the embryonic quail chorioallantoic membrane (CAM) in vivo [19, 32]. However, B-BG as a scaffold for bone repair also has drawbacks such as inherent brittleness [33]. In addition, the over-high alkaline microenvironment derived from the degradative products of B-BG might bring the negative effect for cells and tissues. There have been a number of studies in the literature focusing on the composites created by PCL and conventional BG, which could bring the advantages of both materials [34–39]. However, the bioactivities of BG/PCL composites were dependent on BG and still not satisfactory because of insufficient osteoinduction of conventional BG as compared with novel B-BG materials. Till now, there is no report about the composite between B-BG and PCL polymers. In the present study, novel B-BG/PCL composites were fabricated for the first time, and consequently the mechanical properties and bioactivity of the composites scaffold could be improved. It was also important to identify the optimal B-BG content of B-BG/PCL composites for further application.

In the present study, we synthesized B-BG particles with melting method and investigated their utility as reinforcement for improving the mechanical properties and biological performance of PCL polymer. The effects of the addition of B-BG materials on mechanical properties and degradation behavior of the PCL polymer were assessed. Importantly, the effects of B-BG addition on osteogenic differentiation and angiogenic factor expression of rBMSCs as well as angiogenic differentiation of HUVECs were systematically evaluated in vitro. The potential applications of B-BG/PCL composites with various B-BG contents (0, 10, 20, 30, and 40 wt.%) for bone regeneration are discussed.

## 2. Materials and Methods

### 2.1. Preparation and Characterization of the B-BG/PCL Composite

**Figure 1:**
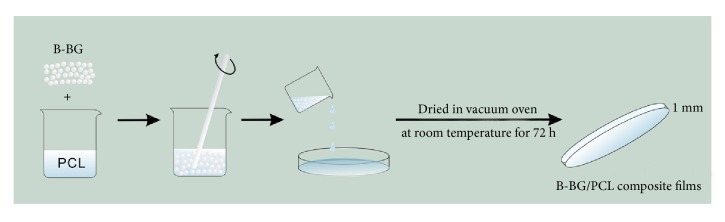
The schematic diagram for the procedure of the B-BG/PCL composite.

The composite films with various B-BG contents (0, 10, 20, 30, and 40 wt.%) were prepared by casting method as shown in Figure 1. Firstly, the B-BG bioactive glass with composition (mol%) of 6Na_2_O, 8K_2_O, 8MgO, 22CaO, 18B_2_O_3_, 36SiO_2_, and 2P_2_O_5_ was prepared using melting method by mixing the required amounts of Na_2_CO_3_, K_2_CO_3_, MgCO_3_, CaCO_3_, H_3_BO_3_, SiO_2_, and NaH_2_PO_4_ (analytical grade, Sinopharm, China), which were melted in a platinum crucible at 1100°C for 1 h. After the melt was quenched in cold steel mould, the glass was crushed, ground, and sieved through 400 mesh nylon screen to obtain the B-BG bioactive glass with particulate size less than 38 *μ*m [40]. The obtained B-BG particulates were used as bioactive component to prepare B-BG/PCL composite films. In which, the PCL (Sigma, USA) was dissolved in dichloromethane with a concentration of 10% (w/v) and a certain amount of B-BG particulates (0, 10, 20, 30, and 40 wt.%, named as PCL, 10wt.%B-BG/PCL, 20wt.%B-BG/PCL, 30wt.%B-BG/PCL, and 40wt.%B-BG/PCL, respectively) were added to PCL solution followed by continuous stirring and ultrasonic dispersion for 30 min to disperse the B-BG particulates homogeneously. Then the mixtures were cast in a glass mould, followed by standing in air for 24 h to evaporate the solvent. After drying in vacuum oven at room temperature for 72 h to eliminate the remaining solvent, the B-BG/PCL composite films with around 1 mm thickness were prepared.

The morphology of the prepared films was observed by scanning electron microscope (SEM, Hitachi SU8220, Japan). The prepared samples were cut into rectangle shape with width 1.0 cm and length 12.0 cm. The rectangle shape samples were used to test tensile strength at the mechanical testing machine (Shimadzu AG- kN, Japan). Three samples from each group were tested to calculate the tensile strength.

The effect of B-BG addition on degradation behavior of PCL polymers was determined by their change of pH values and weight loss percentage in Tris-HCl buffered solution. Firstly, the 0.1 mol/L Tris-HCl buffered solution was prepared by dissolving analytical grade tris(hydroxymethyl) aminomethane in distilled water and then the solution was buffered to pH 7.4 using 1.0 mol/L hydrochloric acid aqueous solution at 37°C. The prepared samples were put into polystyrene bottles containing Tris-HCl buffered solution with the ratio of surface area (cm^2^) to solution volume (cm^3^) of 0.1. For pH value determination, the samples were soaked in Tris-HCl buffered solution without refreshing the soaking medium. At various time points, the pH values of the soaking medium were determined by a pH test-meter (PHS-3C, Leici, Shanghai, China). As for weight loss determination, the Tris-HCl buffered solution was refreshed every 24 h. At the selected time point, the samples were taken out, rinsed with deionized water, and then dried in vacuum at 60°C before further characterization. In the present study, three samples from each group were tested to calculate the pH value and average degradability.

30 wt.% B-BG/PCL composites were soaked in 1 mL medium without FBS and incubated for 4, 7, and 10 days. At each time point, the medium was collected and the concentrations of the boron in medium were measured by inductively coupled plasma atomic emission spectroscopy (ICP-AES; Varian, USA).

### 2.2. Isolation and Culture of rBMSCs

All animal procedures in the present study were approved by the Animal Research Committee of Shanghai Ninth People's Hospital affiliated to Shanghai Jiao Tong University School of Medicine. rBMSCs were obtained from Sprague-Dawley rats at 4 weeks of age as described in our previous study [30]. Briefly, both ends of the femurs were cut off at the metaphyses after rats were sacrificed via overdose of anesthetic. Then, the marrow was flushed out with 10 mL alpha-minimum essential medium (*α*-MEM; Gibco, USA) supplemented with 10% fetal bovine serum (FBS; Gibco, USA) and antibiotics (penicillin 100 U/mL, streptomycin 100 U/mL) using a 22-gauge needle. The primary cells were cultured in an incubator at 37°C and 5% CO_2_ for 4 days, and the medium was renewed every 2 days. Finally, rBMSCs were washed with phosphate-buffered saline (PBS) and passaged using 0.25% trypsin/EDTA when 80% confluence was reached. In the present study, rBMSCs from passages 1 to 2 were applied for the following study.

### 2.3. Cell Adhesion and Proliferation Assay

10 wt.% B-BG/PCL, 20 wt.% B-BG/PCL, 30 wt.% B-BG/PCL, 40 wt.% B-BG/PCL, and pure PCL square samples with the sides of 10 mm and 2 mm thick were sonicated in ethanol and sterilized using ultraviolet light. rBMSCs were seeded on the samples in 24-well plate at a density of 2 ×10^4^ cells/well. Cell adhesion was determined using actin assay as described in our previous study [41]. Briefly, at 6 h after cell seeding, the samples for each group were collected and fixed in 4% paraformaldehyde; and then the samples were treated with 0.1% Triton X-100 and blocked with 1% BSA. The cell actin was labeled with Phalloidin-TRITC (Sigma, USA) while the cell nuclei were contrast-labeled by 40,6-diamidino-2-phenylindole dihydrochloride (DAPI, Sigma, USA). Finally, the actin cytoskeletons for each sample were detected using a confocal laser scanning microscope (CLSM, Leica, Germany). Cell proliferation was determined using the MTT assay after incubation for 1, 4, and 7 days, with the medium replaced every 2 days. Briefly, composite-cell constructs were placed in new wells with 400 *μ*L medium containing 40 *μ*L MTT and incubated in a humidified atmosphere at 37°C for 4 h. Finally, DMSO was used to stop the reaction, and the absorbance of the solution was measured at 490 nm using an ELx Ultra Microplate Reader. Three specimens were tested for each incubation period, and each test was performed in triplicate. Results are reported as OD units. Pure PCL samples were treated as control group.

### 2.4. Alkaline Phosphatase Staining and Quantitative Analysis

rBMSCs were seeded on 10 wt.% B-BG/PCL, 20 wt.% B-BG/PCL, 30 wt.% B-BG/PCL, 40 wt.% B-BG/PCL, and pure PCL samples in 24-well plates at a density of 2 ×10^4^ cells/well and cultured in the medium without mineral or angiogenic inducing components for 10 days. Alkaline phosphatase (ALP) staining was performed according to the manufacturer's instruction (Beyotime, China). Moreover, ALP activity for the cells seeded on the samples was quantitatively determined on days 4, 7, and 10 as described in our previous studies [41, 42]. Briefly, the cells of each sample were incubated with 400 *μ*L lysis buffer at 37°C for 4 hours and then collected after vibration for 30 min. ALP activity was determined by absorbance at 405 nm (BioTek, USA) using p-nitrophenyl phosphate disodium (p-NPP) as the substrate and calculated with reference to a standard product. In the meantime, the total cellular protein content was measured using the Bio-Rad protein assay kit (Bio-Rad, USA) according to the manufacturer's instruction. Finally, the ALP activity was indicated as pNP (mM) per milligram of total cellular protein. All experiments were performed in triplicate.

### 2.5. Quantitative Real-Time PCR

rBMSCs were seeded on 10 wt.% B-BG/PCL, 20 wt.% B-BG/PCL, 30 wt.% B-BG/PCL, 40 wt.% B-BG/PCL, and pure PCL samples in 24-well plates at a density of 2 ×10^4^ cells/well and cultured in the medium without mineral or angiogenic inducing components. On days 4, 7, and 10, the cells cultured on samples were harvested using Trizol (Invitrogen, USA) and RNA was extracted from the Trizol mixture with chloroform, isopropanol, and ethanol. The kit PrimeScript™ Master Mix (Takara, Japan) was used to reversely transcribe 1000 ng of RNA to complementary DNA (cDNA). Finally, the SYBR Green kit (Takara, Japan) was applied to run a quantitative real-time PCR assay on an ABI Q6 real-time quantitative PCR instrument (ABI, USA). The gene expression of osteogenic and angiogenic markers including runt-related transcription factor 2 (RUNX2), bone morphogenetic protein 2 (BMP-2), collagen type 1 (COL1), bone sialoprotein (BSP), osteopontin (OPN), osteocalcin (OCN), vascular endothelial growth factor (VEGF), and angiopoietin-1 (ANG-1) was evaluated, while glyceraldehyde-3-phosphate dehydrogenase (GAPDH) was used as a housekeeping gene for normalization. The primer sequences for each gene are listed in Table 1. All experiments were performed in triplicate.

### 2.6. In Vitro Angiogenesis Assay

In order to evaluate the effect of B-BG/PCL on angiogenesis in vitro, the human endothelial cell line EA.hy926 was cultured on 10 wt.% B-BG/PCL, 20 wt.% B-BG/PCL, 30 wt.% B-BG/PCL, 40 wt.% B-BG/PCL, and pure PCL samples for 4, 7, and 10 days. At each time point, the RNA was extracted and transcribed to cDNA, and quantitative real-time PCR for CD31, CD144, VEGF, and VEGF receptor-2 (KDR) was performed as described previously. In the meantime, actin was used as an internal control for normalization. The primer sequences for each gene are listed in Table 2. All experiments were performed in triplicate.

### 2.7. Statistical Analysis

All means and standard deviations were calculated. Statistical significance between groups was calculated according to ANOVO with SPSS software (SPSS, Chicago, USA). The value of* p*<0.05 was considered statistically significant.

## 3. Results

### 3.1. Characterization of B-BG/PCL Composites

**Figure 2:**
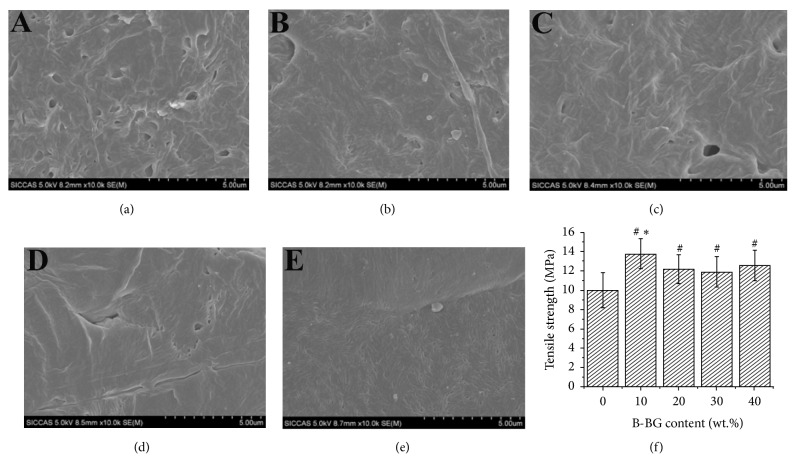
SEM images of control PCL (a) and B-BG/PCL groups ((b) 10 wt.% B-BG/PCL; (c) 20 wt.% B-BG/PCL; (d) 30 wt.% B-BG/PCL; (e) 40 wt.% B-BG/PCL). (f) Tensile strength test for control PCL and B-BG/PCL groups. #P<0.05 indicates the B-BG/PCL groups vs the control PCL group; *∗*P<0.05 indicates the 10 wt.% B-BG/PCL group vs the other B-BG/PCL groups.

**Figure 3:**
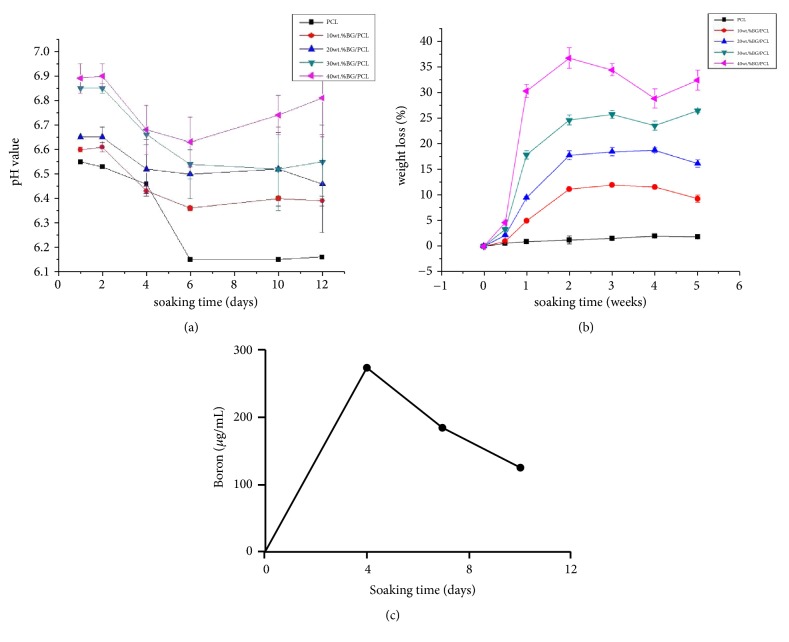
The pH value (a) and weight loss rate (b) tests for control PCL and B-BG/PCL materials immersed in the Tris-HCl buffer solution. (c) The release curve of the boron for 30 wt.% B-BG/PCL composites soaked in 1 mL medium for 4, 7, and 10 days.

The morphology of the prepared films with different amounts of B-BG contents could be observed in Figure 2. The results showed that a few micropores around 0.1-0.5 *μ*m were present in the pure PCL film (Figure 2(a)). The incorporation of BG in the range of 10 and 30 wt.% increased the density in PCL matrix. Further increasing the incorporation amount of BG to 40 wt.%, a large amount of micropores around 0.1-0.2 *μ*m homogenously dispersed of B-BG particles in PCL matrix. In addition, the sample with 10 wt.% BG showed the densest morphology. Most importantly, the BG particles homogenously dispersed among the PCL matrix (Figures 2(b)–2(e)). As shown in Figure 3, the tensile strength of all B-BG/PCL groups was significantly higher than that of the control group; moreover, there was also significant difference between 10 wt.% B-BG/PCL group and the other B-BG/PCL groups (P <0.05).

The pH of all groups immersed in the Tris-HCl buffer solution was significantly fluctuating, showing a decreasing trend in the first 6 days; then the value of pH gradually became relatively stable. The less decrease of pH was detected in all B-BG/PCL groups as compared with the control group. The pH of the control group had dropped to 6.15 after 12 days of immersion, while the range of pH for B-BG/PCL groups was 6.4-6.82 after soaking for 12 days. More importantly, with the increase of B-BG content, the pH value of B-BG/PCL groups was more close to the human neutral value (Figure 3(a)). The results of the degradation rate of materials immersed in Tris-HCl solution showed that the weight loss rate of material degradation changed greatly in the first week. With the increase of the B-BG content, the materials degradated more faster. After the first 2-3 weeks, the value of weight loss rate is close to the value of B-BG content in B-BG/PCL groups, respectively, and the value changed little later. After 5 weeks of degradation, the weight loss rate of 20 wt.% B-BG/PCL and 30 wt.% B-BG/PCL groups reached 16.4% and 25.8%, respectively (Figure 3(b)).

The release curve of the boron showed that 30 wt.% B-BG/PCL composites could release boron throughout the whole observation time, and the concentrations of released boron became lower with time (Figure 3(c)).

### 3.2. Cell Adhesion and Proliferation Assay

**Figure 4:**
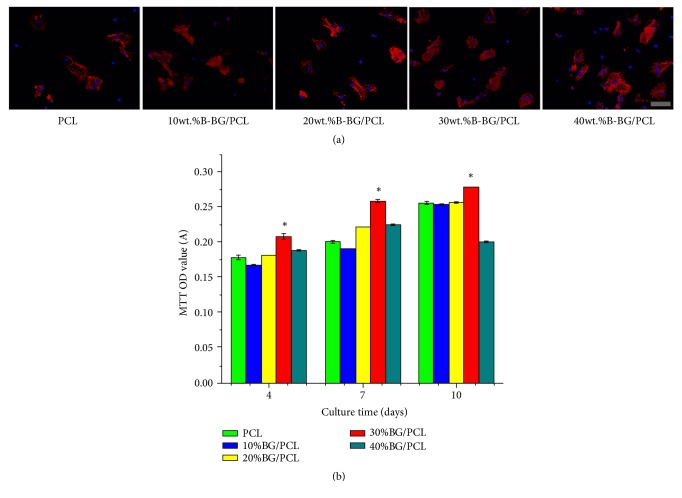
Cell adhesion and proliferation assay. (a) Confocal microscopy images of rBMSCs cultured on control PCL and B-BG/PCL materials for 6 h. (b) MTT assay for rBMSCs cultured on control PCL and B-BG/PCL groups for 4, 7, and 10 days. #P<0.05 indicates the B-BG/PCL groups vs the control PCL group. Scale bar=100 *μ*m.

As shown in Figure 4(a), the cells seeded on PCL spread slightly, almost with a rounded shape, while the cells seeded on B-BG/PCL composites spread well and the actin microfilament system ranged parallel to the long axis of the cells, especially for 30 wt.% and 40 wt.% B-BG/PCL composites. The results of MTT assay showed that the activity of cell proliferation increased with the content of B-BG in B-BG/PCL composites and reached the peak in the 30 wt.% B-BG/PCL group (P<0.05). In the meantime, when the content of B-BG in B-BG/PCL composites reached to 40%, the cell proliferation was decreased and there was significant difference between 40 wt.% B-BG/PCL group and control PCL group at 7 days (P <0.05). The results suggested that the 30 wt.% B-BG/PCL group had the most significant effect on cell proliferation as compared with other groups (Figure 4(b)).

### 3.3. ALP Activity Assay

**Figure 5:**
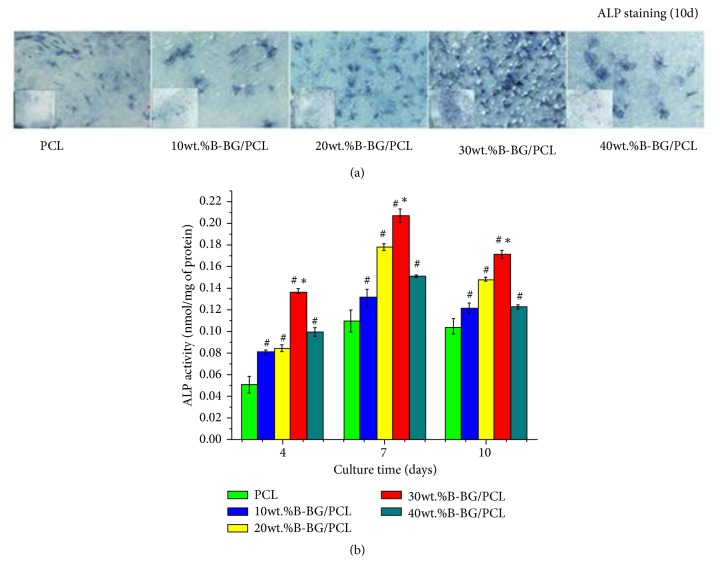
ALP activity assay. (a) ALP staining for rBMSCs cultured on control PCL and B-BG/PCL groups for 10 days. (b) ALP activity quantitative analysis for rBMSCs cultured on control PCL and B-BG/PCL groups for 4, 7, and 10 days. #P<0.05 indicates the B-BG/PCL groups vs the control PCL group; *∗*P<0.05 indicates the 30 wt.% B-BG/PCL group vs the other B-BG/PCL groups.

As shown in Figure 5, ALP staining was more intensive in B-BG/PCL groups as compared with control PCL group, while 30 wt.% B-BG/PCL group processed the most intensive staining. Further, the results of ALP quantitative analysis confirmed that ALP activity was higher in B-BG/PCL groups as compared with control PCL group on days 4, 7, and 10; there was also significant difference between 30 wt.% B-BG/PCL and the other B-BG/PCL groups (P <0.05).

### 3.4. Quantitative Real-Time PCR Assay

**Figure 6:**
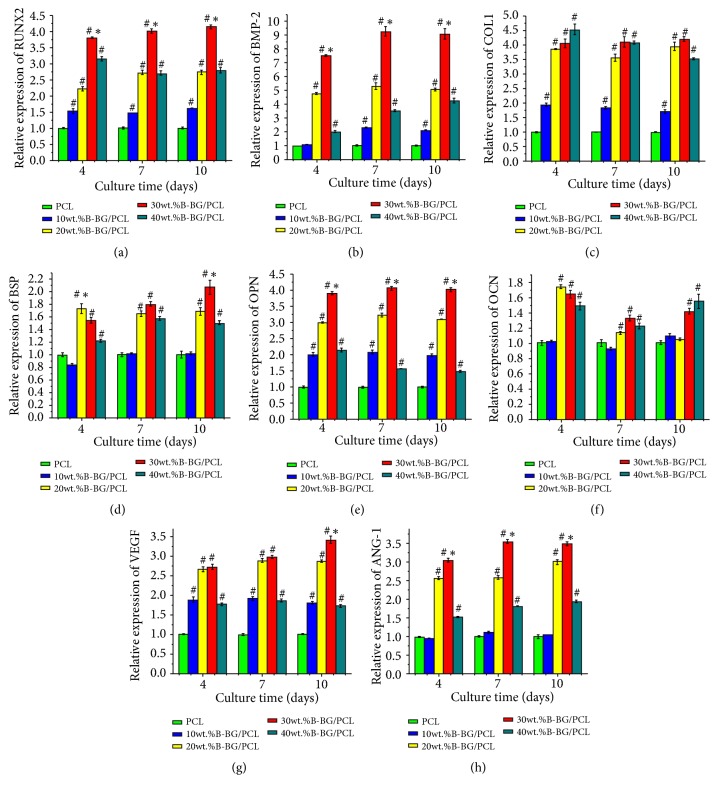
Quantitative real-time PCR assay for rBMSCs cultured on control PCL and B-BG/PCL groups for 4, 7, and 10 days. (a) RUNX2; (b) BMP-2; (c) COL1; (d) BSP; (e) OPN; (f) OCN; (g) VEGF; (h) ANG-1. #P<0.05 indicates the B-BG/PCL groups vs the control PCL group; *∗*P<0.05 indicates the 30 wt.% B-BG/PCL group vs the other B-BG/PCL groups.

In the present study, the osteogenic and angiogenic related markers were evaluated by quantitative real-time PCR (Figure 6). The results showed that all B-BG/PCL groups could enhance the expression of RUNX2, BMP-2, COL1, OPN, and VEGF on days 4, 7, and 10, except for BMP-2 of 10 wt.% B-BG/PCL group on day 4, while 20 wt.% B-BG/PCL, 30 wt.% B-BG/PCL, and 40 wt.% B-BG/PCL groups could promote the expression of BSP, OCN, and ANG-1 on days 4, 7, and 10 (P <0.05). More importantly, 30 wt.% B-BG/PCL group could process most stimulatory effect on the expression of osteogenic and angiogenic related markers as compared with the other B-BG/PCL groups (P <0.05).

### 3.5. In Vitro Angiogenesis Assay

**Figure 7:**
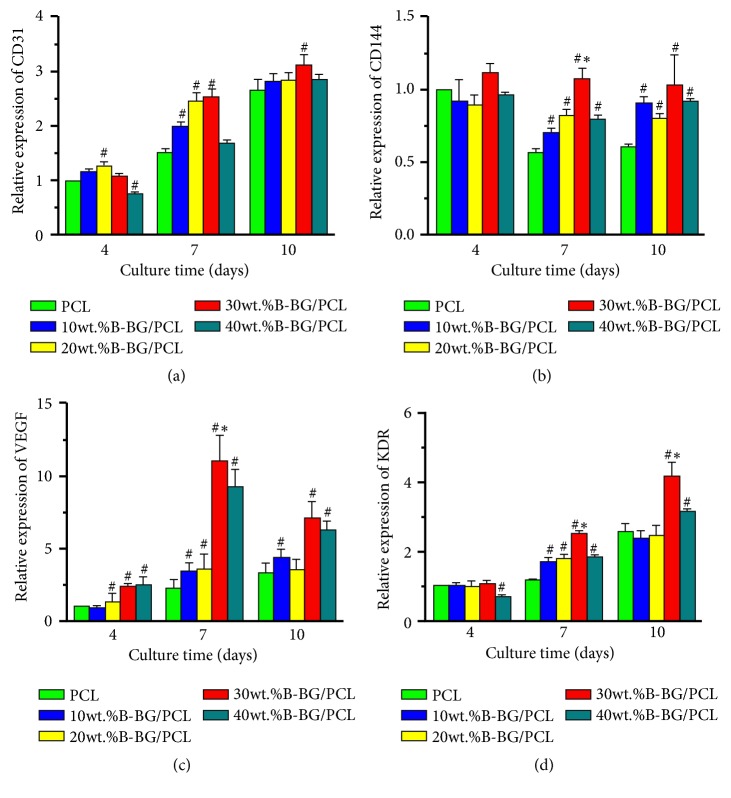
Quantitative real-time PCR assay for EA.hy926 cells cultured on control PCL and B-BG/PCL groups for 4, 7, and 10 days. (a) CD31; (b) CD144; (c) VEGF; (d) KDR. #P<0.05 indicates the B-BG/PCL groups vs the control PCL group; *∗*P<0.05 indicates the 30 wt.% B-BG/PCL group vs the other B-BG/PCL groups.

In order to evaluate the effect of B-BG/PCL on angiogenesis of EA.hy926 cells in vitro, real-time PCR was processed for the expression of CD31, CD144, VEGF, and KDR. As shown in Figure 7, 30 wt.% B-BG/PCL group could enhance the expression of CD31 as compared with control PCL group on days 4 and 10, while 10 wt.% B-BG/PCL, 20 wt.% B-BG/PCL, and 30 wt.% B-BG/PCL groups could promote the expression of CD31 on day 7 (P <0.05). All B-BG/PCL groups could enhance the expression of CD144 as compared with control group on days 7 and 10, while 30 wt.% B-BG/PCL groups achieved the highest value on day 7 (P <0.05). Moreover, all B-BG/PCL groups could enhance the expression of VEGF on days 4, 7, and 10, expect for 10 wt.% B-BG/PCL group on day 4 and 20 wt.% B-BG/PCL group on day 10. As for the expression of KDR, there was significant difference between all B-BG/PCL groups and control group on day 7, while there was also significant difference between 30 wt.% B-BG/PCL group and control group on day 4 and between 30 wt.% B-BG/PCL, 40 wt.% B-BG/PCL groups, and control group on day 10. Importantly, 30 wt.% B-BG/PCL groups achieved the highest value on days 7 and 10 (P <0.05).

## 4. Discussion

PCL is one of the most extensively investigated polymers for bone regeneration due to its interesting and attractive set of properties, such as low melting point (~60°C) and glass transition temperature (~−60°C) and high toughness [37]. However, the main limitation of PCL is its relatively slow bioresorption rate in vivo [37, 43]. Moreover, the products of PCL degradation are acidic and could cause delayed aseptic inflammatory response when implanted in vivo, resulting in failure of implantation [44, 45]. The purpose of the present study is to improve the mechanical properties and degradation properties of PCL materials by B-BG composite with the contents of 0, 10, 20, 30, and 40 wt.%, respectively.

In the present study, combining B-BG with pure PCL polymers could improve the tensile strength of the materials, while the tensile strength of 10 wt.% B-BG/PCL group was higher than that of all other groups, which was almost 37.98% higher than that of the control group. The higher mechanical strength of 10 wt.% B-BG/PCL was due to the highest densification in this group. Importantly, the results of the pH value for material soaked in Tris-HCl solution showed pH value was weakly acidic and the fluctuation was obvious, showing a general downward trend in the first 6 days. This phenomenon might be related to the release of acid ions such as SiO_3_^2−^, HSiO_3_^−^, or SiO_4_^4−^ and the temperature. After the first 6 days, the pH value tended to be relatively stable with slightly rising and, more importantly, the decrease of pH value in B-BG/PCL groups was less than that of the control group. After 12 days of immersion, the pH value of the control group was decreased to 6.15, while the pH value of B-BG/PCL groups was between 6.4 and 6.82, respectively. Interestingly, the pH value of B-BG/PCL groups was more close to the human neutral value with the increase of B-BG content, which might be attributed to different content of silicate and borate in B-BG/PCL groups [46].

The previous studies reported that the pure PCL material was stable and slowly degradated in vitro, and the total time for complete degradation of PCL was about 2-3 years, which was not conducive to the guidance of the new bone formation [47]. In the present study, the results showed that the degradation trend of the pure PCL group was slow and stable, while the degradation rate of B-BG/PCL groups was larger in the first week. It was also shown that the faster degradation was achieved with the increase of the B-BG content, and weight loss rate of B-BG/PCL groups almost reached B-BG content after 2-3 weeks. It is suggested that the degradation of B-BG/PCL composites was mainly determined by B-BG content. Besides, the weight loss rate of B-BG/PCL composites began to decrease after 2-3 weeks of immersion, which could be due to the formation of apatite crystals on the B-BG/PCL surface [48]. After 5 weeks of degradation, the weight loss rate of 40 wt.% B-BG/PCL group reached 32.6%; this fast degradation rate might reduce the mechanical properties of materials and provide insufficient guiding effect on new bone formation. However, After 5 weeks of degradation, the weight loss rate of 20 wt.% B-BG/PCL and 30 wt.% B-BG/PCL groups reached 16.4% and 25.8%, respectively, which might meet the requirements of bone repairing under different degradation rate. Based on these results, it is suggested that the degradation performance of 20 wt.% B-BG/PCL and 30 wt.% B-BG/PCL groups was more suitable than the other groups; besides, the tensile strength of 20 wt.% B-BG/PCL and 30 wt.% B-BG/PCL groups was better than the control PCL group, being only worse than 10 wt.% B-BG/PCL group.

It is well known to us that pure PCL polymers lack biological activity, osteoconductivity, and osteoinduction. In the present study, B-BG inorganic substances were combined with PCL materials in order to enhance biological performance of PCL. The results of cell actin and MTT assay showed that 30 wt.% B-BG/PCL group had more pronounced promoting effect on the cell attachment and proliferation of BMSCs as compared with PCL group. More importantly, ALP is an early marker of osteogenic differentiation and has important effect on cell differentiation and maturation [49]. The results of ALP staining and qualitative showed that the addition of B-BG to PCL could enhance ALP activity of rBMSCs, this enhanced effect was related to the addition of B-BG content, and 30 wt.% B-BG/PCL group had the most significant effect. Real-time PCR assay was further applied to evaluate gene expression of osteogenesis-related markers. Runx2, BMP-2, and COL1 are chosen as the markers for early and middle stage of osteogenic differentiation [50], while BSP and OCN are markers of late stage. Besides, OPN is considered to be an indicator from early to late stage [51]. The results showed that B-BG/PCL could promote the expression of these genes in the early, middle, and late stages of osteogenic differentiation in B-BG content-dependent manner, and 30 wt.% B-BG/PCL group had significantly higher level than the other groups.

Vascularization of the implanted material is the basis in the process of repairing bone defects; moreover, the amount of new bone formation is closely related to the degree of vascularization [52]. Previous study reported that boric acid could induce the protein expression of VEGF in vitro [20]. It was also reported that oral boric acid could promote formation of properly organized vascular bundles in rat models of Achilles tendons [53]. Recent studies also reported that boric doped BG (B-BG) materials could stimulate cell proliferation, migration, and tube formation of HUVECs and angiogenesis on the vasculature of the embryonic quail CAM in vivo [19, 32]. In the present study, the effects of novel B-BG/PCL composites on the expression of angiogenic factors of rBMSCs and angiogenic differentiation of HUVECs were evaluated. The results showed that B-BG/PCL composites could enhance the expression of VEGF and ANG-1 of rBMSCs, while 30 wt.% B-BG/PCL composites achieved the best stimulatory effect. VEGF is considered as a key angiogenic factor, which processes the strongest and most significant biological activity in enhancing blood formation [54, 55]. ANG-1 plays an important role in the late stage of blood vessel formation, including the stabilization of the endothelial sprout and its interaction with pericytes [56]. It is suggested that the enhanced expression of VEGF and ANG-1 of rBMSCs could take the paracrine effects on host angiogenic cells and consequently promote angiogenesis in vivo. Besides, B-BG/PCL composites could also promote angiogenic differentiation of HUVECs, such as enhanced expression of vascular endothelial cell markers (CD31 and CD144) [57, 58], VEGF, and VEGF receptor-2 (KDR), while 30 wt.% B-BG/PCL composites possessed the best ability. However, the effects of the novel B-BG/PCL composites on osteogenesis and angiogenesis need to be verified in critical-sized bone defect animal models in the future.

## 5. Conclusion

In the present study, B-BG containing PCL materials have more suitable mechanical properties, pH value, and degradation rate as compared with pure PCL. More importantly, B-BG/PCL composites could promote cell proliferation, osteogenic differentiation, and expression of angiogenetic genes of rBMSCs, while enhancing angiogenetic differentiation of HUVECs. These enhanced effects had concentration dependence of B-BG content, while 30 wt.% B-BG/PCL composites achieved the greatest stimulatory effect. It is suggested that 30 wt.% B-BG/PCL composites might be novel promising biomaterials for bone regeneration.

## Figures and Tables

**Table 1:** Primer sequences used for rat BMSCs.

Gene	Primers (F=forward; R=reverse)	Accession	Product
		numbers	size (bp)
RUNX2	F: 5′ATCCAGCCACCTTCACTTACACC3′	NM_053470.2	199
	R: 5′GGGACCATTGGGAACTGATAGG3′		

BMP-2	F: 5′GAAGCCAGGTGTCTCCAAGAG3′	NM_017178.1	122
	R: 5′GTGGATGTCCTTTACCGTCGT3′		

COL1	F: 5′CTGCCCAGAAGAATATGTATCACC3′	NM_053304.1	198
	R: 5′ GAAGCAAAGTTTCCTCCAAGACC3′		

BSP	F: 5′AGAAAGAGCAGCACGGTTGAGT3′	NM_012587.2	175
	R: 5′ GACCCTCGTAGCCTTCATAGCC3′		

OPN	F: 5′CCAAGCGTGGAAACACACAGCC3′	NM_012881.2	165
	R: 5′GGCTTTGGAACTCGCCTGACTG3′		

OCN	F: 5′CAGTAAGGTGGTGAATAGACTCCG3′	NM_013414.1	172
	R: 5′GGTGCCATAGATGCGCTTG3′		

VEGF	F: 5′GGCTCTGAAACCATGAACTTTCT3′	NM_001110334.1	165
	R: 5′GCAGTAGCTGCGCTGGTAGAC3′		

ANG-1	F: 5′GGACAGCAGGCAAACAGAGCAGC3′	NM_053546.1	130
	R: 5′CCACAGGCATCAAACCACCAACC3′		

GAPDH	F: 5′CCTGCACCACCAACTGCTTA3′	NM_017008.4	120
	R: 5′GGCCATCCACAGTCTTCTGAG3′		

**Table 2:** Primer sequences used for EA.hy926 cells.

Gene	Primers (F=forward; R=reverse)	Accession	Product
		numbers	size (bp)
RUNX2	F: 5′ATCCAGCCACCTTCACTTACACC3′	NM_053470.2	199
	R: 5′GGGACCATTGGGAACTGATAGG3′		

BMP-2	F: 5′GAAGCCAGGTGTCTCCAAGAG3′	NM_017178.1	122
	R: 5′GTGGATGTCCTTTACCGTCGT3′		

COL1	F: 5′CTGCCCAGAAGAATATGTATCACC3′	NM_053304.1	198
	R: 5′ GAAGCAAAGTTTCCTCCAAGACC3′		

BSP	F: 5′AGAAAGAGCAGCACGGTTGAGT3′	NM_012587.2	175
	R: 5′ GACCCTCGTAGCCTTCATAGCC3′		

OPN	F: 5′CCAAGCGTGGAAACACACAGCC3′	NM_012881.2	165
	R: 5′GGCTTTGGAACTCGCCTGACTG3′		

## Data Availability

The data (Figures 1–6) used to support the findings of this study are included within the article.
